# Utilization of Primary and Secondary Medical Care among Disadvantaged Populations: A Log-Linear Model Analysis

**DOI:** 10.5539/gjhs.v6n5p9

**Published:** 2014-04-27

**Authors:** Gregory Yom Din, Zinaida Zugman, Khashper Alla

**Affiliations:** 1 The Open University of Israel, Raanana, Israel; 2 Faculty of Exact Sciences, Tel-Aviv University, Tel-Aviv, Israel; 3 Independent Consultant on Data Analysis, Kazrin, Israel; 4 McGill University Health Center, Montreal, Canada

**Keywords:** medical care utilization, disadvantaged populations, equity, log-linear models

## Abstract

**Aim:**

We examined how, where an overall population is covered by universal health insurance, characteristics of disadvantaged populations interact to influence inequality in primary and secondary medical care utilization.

**Subject and Methods:**

Disadvantaged populations, the focus of the study, were defined as populations who have lower socio-economic status (SES), who are elderly and/or reside in a peripheral area. Data from the 2009 Israeli National Health Survey were analysed using log-linear models to estimate utilization of medical care.

**Results:**

The main findings were: a) pro-poor utilization of primary medical care among elderly populations, with higher odds ratios for low SES populations in the periphery; (b) lack of interaction between SES and primary medical care utilization among younger populations, between SES and secondary medical care utilization among the elderly and pro-rich utilization of secondary medical care among younger populations who did not regularly visit general practitioners (GP); (c) the odds ratios of secondary medical care utilization increased as SES decreased for both elderly and younger populations who also regularly visited a GP.

**Conclusion:**

Potential policy implications for disadvantaged populations, regarding possible inequality in primary and secondary medical care utilization, can be drawn using log-linear model analysis of interactions among characteristics (SES, age, location) of disadvantaged populations.

## 1. Introduction

Disadvantaged populations are defined as those with a relative deprivation of social and financial resources and/or occupational prestige. For these populations, we can often see a violation of the principle of equity in health care, defined as “equal utilization for equal need” (Aday & Andersen, 1981; Whitehead, 1992). The use of health care can be considerably disadvantaged by the travel distances (Jordan et al., 2004). The possibility that older people, not necessarily socioeconomically disadvantaged, may experience particularly poor availability of health care is noted by Asthana and Gibson (2008).

A number of recent empirical studies have examined the effects of various factors and their interactions characterizing inequality in medical care utilization: accessibility within the periphery (Mangano, 2010), SES (Kim et al., 2012), insurance status (Wendt, 2009), age, gender, income, education level (Hansen et al., 2012), and regional variation (Masseria & Giannoni, 2010).

The purpose of this study was to examine how the aforementioned interactions among characteristics of disadvantaged populations influence primary and secondary medical care utilization, under the assumption that the overall population is covered by universal health insurance, as required by Israeli law.

In Israel, all permanent residents are insured for basic medical services under the National Health Insurance Law (Filc, 2010; Rosen & Samuel, 2009), and close to 80 percent of the population has some form of supplementary insurance (Van de Ven et al., 2013). The mandatory health insurance is financed by a progressive health tax and provided by one of four health funds, operating as insurers and providers. As members of these funds, residents receive free primary care and hospitalization services (one of the funds charges a small co-payment for primary care visits) while specialty care and imaging services incur a copayment (Shadmi et al., 2011). However, a number of authors have reported considerable disparities in medical care utilization and have attributed the differences to low SES (Monnickendam et al., 2007), difficulty in accessing medical care in peripheral regions (Lubetzky et al., 2011), cultural and educational differences (Benyamini et al., 2008) and ethnicity (Baron-Epel et al., 2007). Inequality in utilization of different kinds of medical care in Israel (primary care visits, medical specialist visits, diagnostic tests, hospitalizations) was shown by Shadmi et al. (2011). Elderly people living in the periphery warrant special attention, because of documented obstacles in accessibility and affordability of specialist services (Iecovich & Carmel, 2009).

However, statistical evidence of interactions between factors affecting inequality in health care utilization is still limited, and continues to be the focus of studies from Israel and other countries with publicly funded health care systems, such as Canada (Alter et al., 2011) and Norway (Hansen et al., 2012). In many articles, regression techniques are used to model medical care utilization as a function of health, socio-economic and demographic factors, to measure socio-economic inequalities and unmet needs in the distribution of medical care utilization. In the case of categorical data, in social sciences log-linear modeling for contingency tables is widely used to analyze interactions among factors (Agresti, 2002), particularly in healthcare applications (Brennan et al., 2012; Bylund et al., 2010). In this study, these models were utilized to focus on interactions between several categories of disadvantage factors, not only on the marginal effects of single factors on the response variable. The factors used in contingency tables were considered as independent categorical variables. The response measure was presented by frequencies in cells of the contingency tables, showing use of different types of medical care for a given combination of disadvantaged population categories.

Initially, the utilization of primary and secondary medical care among groups differentiated by location, age and SES was analyzed. Then, an expanded model was employed which allowed secondary medical care utilization to be estimated separately for populations who visited or did not visit a general practitioner. Contingency tables were calculated to select the corresponding, best-fit log-linear models, which enabled the significance of disadvantage factors and their interactions to be calculated. The estimated log-ratios of medical care utilization for various groups of disadvantaged populations were interpreted as indicators of health care inequality among these groups.

## 2. Data and Methods

### 2.1 Data Source

The data for this study was obtained from the Israeli National Health Survey conducted from January to December 2009. The survey questionnaire was designed to match OECD data requirements and was distributed in Hebrew, English, Russian and Arabic. The data was collected from 28,968 members of 8,713 households. For every respondent, his/her relationship to the head of household was recorded.

The number and percentage of total respondents for various categories – location, gender, age and years of schooling – are shown in Table 1. The demographic profile of the population in the center of the country and in the periphery was clearly different in most categories. In the central regions, the elderly population (defined as those over 60 years old) comprised 16.7% of the total population. In the periphery, 11.7% of the population was elderly, an appreciably lower value. Among the elderly living in the central regions, 55.9% were women, while in the periphery the percentage of elderly women was lower at 52.2%. Heads of households in the central regions showed approximately 9.7% more years of schooling than those in the periphery.

**Table 1:** Characteristics of respondents in the Israeli National Health Survey, 2009

Group of respondents	Location							
	Central		Intermediate		Peripheral		National	

	respondents	%	respondents	%	respondents	%	respondents	%
Total respondents	15,493	100%	8,897	100%	4,572	100.0%	28,962	100%
Men	7,513	48.5%	4,362	49.0%	2,288	50.0%	14,163	48.9%
Women	7,980	51.5%	4,535	51.0%	2,284	50.0%	14,799	51.1%
*Age ≥60*	2,586	16.7%	1,220	13.7%	534	11.7%	4,340	15.0%
Men	1,141	7.4%	555	6.2%	255	5.6%	1,951	6.7%
Women	1,445	9.3%	665	7.5%	279	6.1%	2,389	8.2%
*Age <60*	12,913	83.3%	7,677	86.3%	4,038	88.3%	24,628	85.0%
Men	6,372	41.1%	3,807	42.8%	2,033	44.5%	12,212	42.2%
Women	6,535	42.2%	3,870	43.5%	2,005	43.9%	12,410	42.8%
Years of schooling, heads of household	13.8		13.2		12.6		13.5	

In this study, primary medical care is defined as treatment and preventive measures provided by a range of practitioners, including GP, pediatricians and internal physicians (for our purposes, GP equates with any of these medical care providers). Secondary medical care includes services provided by specialist doctors (SD), often arranged through referrals or consultations after a preliminary evaluation by a family doctor. Specialists include gynecologists, orthopedists, ear-nose-throat specialists, ophthalmologists, cardiologists, surgeons, urologists, neurologists, pulmonologists, psychiatrists and other specialized medical practitioners. This categorization of medical care has been used in many recent publications that review national health care systems (Rosen & Samuel, 2009; Strandberg-Larsen et al., 2007). Approximately 13% of the respondents visited GP in the two weeks prior to interview, and 6% visited SD in the same period.

The following data was used for calculation of SES: occupation, years of schooling, socio-economic cluster and the size of family residence. A summary of the survey data used in the statistical analysis is shown in Table 2.

**Table 2:** Data used in the log-linear models

**Name**	**Description and values of the variable in the survey**	**Categorized values in the statistical analysis**
Primary medical care	Number of visits to GP doctors in the last two weeks	Yes (visited), no (not visited)
Secondary medical care	Number of visits to specialist doctors in the last two weeks	Yes (visited), no (not visited)
Location	Peripheral index combining the region’s proximity to other areas, adjusted for their population size and the proximity to Tel Aviv, the business heart of the country. Registered values are 1, 2, 3	Peripheral, intermediate, central

**Age**	**20 categories of age ranging from 0 to 85+**	**Age≥60, age<60**
Data used for calculation of SES:		
Occupation	11 possible answers sorted by skill level from 1 (does not work) to 11 (academic professionals)	Five ordered categories: 1, 2, 3, 4, 5
Years of education	Range of 7 possible answers from 0 (never studied) to 6 (16+ years)	Five ordered categories: 1, 2, 3, 4, 5
Socio-economic cluster	Belonging to one of ten clusters in which municipalities in Israel are classified	Five ordered categories: 1, 2, 3, 4, 5
Number of rooms in the household	From 0.5 to 9.5 rooms	Five ordered categories: 1, 2, 3, 4, 5

The socio-economic clusters (SEC) used by the Israel Central Bureau of Statistics to classify municipalities or regional councils are created using an average of per capita income, the percentage of vehicle owners among the population and other characteristics (Afriat & Dahan, 2010). These area-based measures are used by researchers to monitor and assess socio-economic inequalities in health (Vernez Moudon et al., 2011).

### 2.2 Calculation of SES

The socio-economic status of the respondents is determined by income, wealth and education of its members, and refers to their position within a social hierarchy and access to goods and services (Krieger et al., 1997). In many empirical studies, SES define social disadvantage (Pawils et al., 2012).

To prepare input data for the calculation of SES, the corresponding data from Table 2 (occupation, education and SEC) were grouped in such a way that every item was assigned, with a frequency distribution large The socio-economic clusters (SEC) used by the Israel Central Bureau of Statistics to classify municipalities or regional councils are created using an average of per capita income, the percentage of vehicle owners among the population and other characteristics (Afriat & Dahan, 2010). These area-based measures are used by researchers to monitor and assess socio-economic inequalities in health (Vernez Moudon et al., 2011), enough to be of use, integer values ranging from 1 to 5. The fourth item (related to number of rooms in the household) was calculated with the following expression: ln(*number of rooms* · *SEC*), weighting *number of rooms* by municipal data and using logarithms in order to arrive at a good approximation of normal distribution, adjusted to take values from the same range, 1 to 5. “Education” received a maximal mean value, explained by a high share (88 percent) of respondents with more than 10 years of education (Table 3).

**Table 3:** Description of the SES index items

Items (take values from the range [1, 5])	Mean	Std. Deviation
occupation	2.00	1.41
education	3.46	0.71
SEC	2.59	1.46
ln (number of rooms x SEC) (adjusted)	3.17	0.36

**Figure 1:**
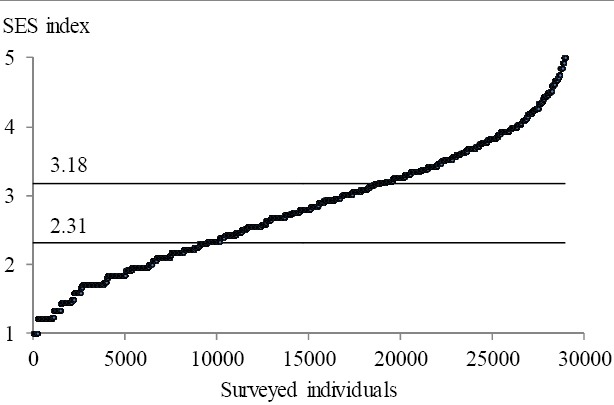
Values of SES and its 33.3 percentiles

SES was defined by an equally-weighted score given to these four categories, and was calculated for every respondent. For the following statistical analysis, the SES values were divided into three equal percentiles that corresponded to low, middle and high levels of respondent SES. The first percentile was equal to 2.31 and the second percentile to 3.18 (Figure 1).

**Figure 2:**
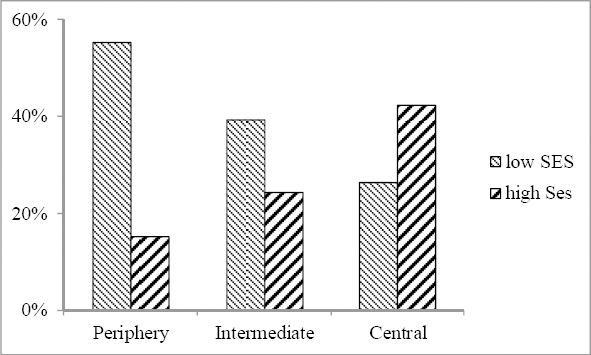
Distribution of respondents with low and high level of SES by location

The distribution of respondents with low and high levels of SES by location showed that in the periphery, 55% of residents had low SES, as compared to 26% in the center. For high SES respondents, the opposite trend could be seen: in the central regions 42% of respondents had high SES, as compared to 15% in the periphery (Figure 2).

A reliability analysis based on the model of Cronbach’s alpha (Cronbach, 1951) was made for the SES characteristics. The alpha, indicating internal data consistency, was estimated as 0.68 for the sample of heads of household and spouses, and as 0.70 based on standardized items for the same sample. For social science applications, alpha equal to 0.70 or higher can be considered “acceptable” indicating moderate consistency between items (Valentine et al., 2011). Thus, internal consistency for the data selected for calculating SES was assumed.

### 2.3 Using Log-Linear Models

Frequency tables were employed for medical care utilization analysis, formed by the following categorical variables:

*L* (location) – number of categories *i* is 3 (periphery, intermediate, central);

*A* (age) – number of categories *j* is 2 (age ≥60, <60);

*SES* (socio-economic status) – number of categories *k* is 3 (low, middle, high);

*Vis* (visited doctors) – number of categories *l* is 2 (visited or not visited, including telephone consultations, in the two week reporting period).

In log-linear models, the natural logarithm of observation distribution is presented as a linear combination of “main effects” and their “interactions.” Let *f_ijkl_* denote the expected frequency for the table cell that corresponds to categories *i*, *j*, *k* and *l* of *L*, *A*, *SES* and *Vis*, respectively. The saturated log-linear model of order 4 is defined as follows:


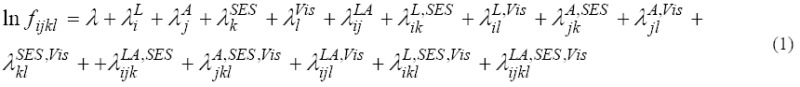


where λ denotes the overall mean of ln *f_ijkl_*,


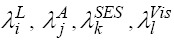
 are the main effects for *L*, *A*, *SES* and *Vis*, respectively,


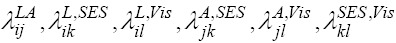
 are the interactions of the second order,


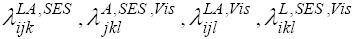
 are the interactions of the third order and


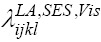
 is the interaction of the fourth order.

The parameters of the log-linear model in the right side of (1) are interpreted as the odds of a variable being in a specific category, rather than in the reference category (the main effect of the variable), odd ratios (two-way interaction effects), ratios of two odd ratios (three-way interaction effects) and so on. Usually, the last category of a variable is selected as the reference category.

The model (1) is a saturated model because it contains all the possible interactions among the variables *L*, *A*, *SES*, and *Vis*: 16 main effects and interactions of 4 orders. Saturated models are considered to be special cases among hierarchical models. The latter are frequently used in socio-economic applications, due to the simplicity in selecting best-fit models. For hierarchical models, including the interaction of a higher order group of variables leads to the inclusion of all lower order interactions and the main effects for these variables. The highest order variables are termed the “generating class” for the model.

For every frequency table related to the utilization of primary or secondary medical care, a saturated model was chosen as a base model. In order to find a more straightforward model, the effect with the largest significance level for the likelihood of ratio statistic of change in χ^2^ after deletion of this effect, was looked for. If an effect was found with a significance level larger than 0.05, it was removed from the model, which would then be estimated again. Otherwise, the last examined model was left as the best-fit model (Benedetti & Brown, 1978; IBM SPSS Statistics 20; Rosenfeld, 2005). This calculation of the loss of fit resulting from the removal of an effect allowed for an assessment of its importance in the model (Upton, 1991).

Two models of the form (1) were estimated for the utilization of primary medical care (in this case *Vis* denoted visits to GP) and secondary medical care (in which *Vis* denoted visits to SD): Model GP and Model SD, respectively. The structure of the selected best-fit models and an estimation of odds ratios enabled the factors that influence utilization of medical care by disadvantaged populations to be inferred.

For example, 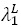 the main effect for Model GP (in which reference category *i* = 3, residence in the center region) was interpreted as the expected odds of living in the periphery as opposed to living in the center. For higher order interactions, an interaction effect, “visited GP to non-visited GP,” comparing low SES to high SES for respondents aged ≥60 and generalizing for all three location categories, was calculated as a ratio of the following odds ratios:


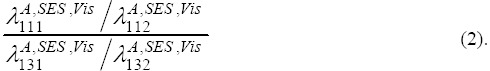


These, in turn, were calculated using the model estimated parameters, under the assumption that these parameters were included in the best-fit selected model. An effect generated by (2) greater than 1 indicated pro-poor utilization (pro-poor defined as in favor of populations with low SES), smaller than 1 indicated pro-rich utilization (accordingly, with a high SES) and close to 1 indicated no influence of SES on the utilization of primary medical care. Formulas and examples of calculating odds ratios and their confidence intervals for high order log-linear models were interpreted in details, as for example in Agresti’s monograph (Agresti 2002, sections 2.2.3, 3.1.1, and 8) and in Kaufman and Schervish’s expository discussion (Kaufman & Schervish, 1987).

The third estimated model, Model SD/GP, included five variables: location, age and SES as in (1) plus two additional variables: *VisGP* (visited GP) and *VisSD* (visited SD). The initial saturated form of this model included all 32 main effects and interactions, enabling the influence of the three “disadvantage” variables on secondary medical care utilization for populations who visited or did not visit a GP to be estimated separately.

## 3. Results

After the survey data was categorized (Tables 1–3, Figures 1, 2), and starting from the initial saturated form (1), the log-linear models were estimated with SPSS (IBM SPSS Statistics 20). Categorical variable frequencies used for coding input data are shown in Table 4. Given the 18 possible combinations of disadvantaged population categories (drawn from 3 for location, 2 for age and 3 for SES) and the 4 possible combinations of visited or not visited GP and SD, this table contains 72 cells.

**Table 4:** Frequency distribution of variables

Location	Age	Visited GP	Visited SD	Low SES		Middle SES		High SES	

				yes	no	yes	no	yes	no
Peripheral	≥60	yes		17	102	6	42	2	9
Peripheral	≥60	no		21	159	16	97	8	55
Peripheral	<60	yes		16	236	11	100	6	58
Peripheral	<60	no		51	1926	53	1028	36	517
Intermediate	≥60	yes		20	120	25	140	10	38
Intermediate	≥60	no		34	292	42	316	18	165
Intermediate	<60	yes		21	300	37	234	13	184
Intermediate	<60	no		61	2659	96	2332	86	1654
Central	≥60	yes		51	219	55	259	30	155
Central	≥60	no		58	443	84	623	71	538
Central	<60	yes		20	315	37	370	56	574
Central	<60	no		96	2892	146	3249	334	4824
Total				466	9663	608	8790	670	8771

### 3.1 Selection of the best-fit hierarchical log-linear model

For Model GP, the best-fit model was selected with the following generating class: 
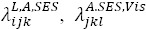
. This model included two interactions among third order factors: location*age*SES and age*SES*visited GP, as well as all lower order interactions and their main effects. In the generating class, visited or did not visit GP interacted with the age and SES of the respondents. There were no interactions between visited GP and location.

For Model SD, the best-fit model was selected with the following generating class: 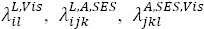. This model included an interaction of the second order, location*visited SD, and two interactions of the third order, location*age*SES and age*SES*visited SD. Visited or not visited SD interacted with location, age and SES. This model did not enable the odds ratio of visited SD specifically for those who visited or did not visit GP to be estimated.

For Model SD/GP, the generating class for the best-fit selected model was determined by: 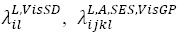 and 
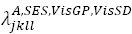
. This model included an interaction of the second order, location*visited SD, and two interactions of the forth order, location*age*SES*visitedGP and age*SES*visitedGP*visitedSD. Unlike Model SD, the Model SD/GP enabled the odds ratio of visited SD for various age groups and levels of SES to be estimated separately for respondents who visited or did not visit GP.

### 3.2 Odds Ratios and Confidence Intervals

For each of the three models, odds ratios relevant to the study questions were calculated, using the odds ratios for the generating class from SPSS output tables (the Analyze-Loglinear module) and the method exemplary shown in (2). In Table 5, the odds ratios of primary medical care utilization for Model GP are shown. In the columns “lower 95% CI” and “upper 95% CI,” bounds of 95% confidence intervals for odds ratios are indicated. The intervals are asymmetric because of skewed sample odds ratios (Agresti, 2002).

**Table 5:** Odds ratios: “visited GP to non-visited” for Model GP, and “visited SD to non-visited” for Model SD

Interaction	Odds ratio	Lower 95% CI	Upper 95% CI
Model GP			
age ≥60			
low SES to high	1.84	1.54	2.20
middle SES to high	1.57	1.32	1.87
age<60			
low SES to high	0.99	0.89	1.09
middle SES to high	0.96	0.86	1.06
age ≥60 vs age<60			
low SES	4.46	3.93	5.05
middle SES	3.91	3.45	4.44
high SES	2.39	2.04	2.80
Model SD			
age ≥60			
low SES to high	1.05	0.83	1.33
middle SES to high	1.08	0.86	1.35
age<60			
low SES to high	0.48	0.41	0.56
middle SES to high	0.78	0.68	0.90
age ≥60 to age<60			
low SES	4.66	3.85	5.65
middle SES	2.93	2.47	3.49
high SES	2.13	1.75	2.60

For the elderly respondents, the odds ratio of visited GP by residents with low SES to residents with high SES was 1.84 (a confidence interval of 1.54, 2.20). A similar odds ratio, 1.57, was found for residents with middle to high SES. These results indicate pro-poor utilization of primary medical care for elderly respondents. For respondents younger than 60 years old, the corresponding odds ratios for those with different SES are close to 1, indicating no interaction between the SES of younger respondents and their utilization of primary medical care. The increase in odds ratios of visited GP for elderly relative to younger respondents is significantly greater for respondents with low and middle SES (4.5 and 3.9, respectively), than for those with low SES (2.4 only).

The odds ratios of secondary medical care utilization for Model SD are presented in the second half of Table 5. For elderly respondents, the odds ratios of visited SD for persons with low or middle SES to persons with high SES were close to 1, indicating no interaction between SES and secondary medical care utilization for this age group. For younger respondents, the corresponding odds ratios of visited SD for respondents with low SES to those with high SES was 0.48, with a SI of 0.41, 0.56. This indicates highly significant pro-rich utilization of secondary medical care for this age group. The odds ratios of visited SD for elderly relative to younger respondents were similar to those shown in the same table for visited GP: the lower the SES, the higher theodds ratio.

Finally, an analysis of visited SD was performed using Model SD/GP when respondents were differentiated by visited/non-visited GP categories (Table 6). For those who visited a GP, the odds ratios of visited SD for respondents with low/middle SES to high SES, for elderly and non-elderly respondents alike, was not significantly different from 1. However, for those who did not visit a GP, the odds ratios for younger respondents were significantly less than 1. Compared with Model SD, this allowed a more specific conclusion on the pro-rich utilization of secondary medical care for younger respondents to be drawn: only those younger respondents that did not visit a GP displayed pro-rich utilization of secondary medical care. Another result of Model SD/GP was that for elderly respondents in the periphery, the odds ratio of visited GP for those with low SES to those with high SES was higher than in center (90% significance).

**Table 6:** Odds ratios “visited SD to non-visited” for Model SD/GP – secondary medical care utilization differentiated by categories “visited/not-visited GP”

Interaction	Odds ratio	Lower 95% CI	Upper 95% CI
visited GP/not visited			
periphery			
age ≥60			
low SES to high^*^	3.81	2.15	6.75
center			
age ≥60			
low SES to high^*^	1.78	1.48	2.15
visited SD/not visited (for those who visited GP)			
age ≥60			
low SES to high	0.97	0.65	1.46
middle SES to high	0.95	0.64	1.43
age<60			
low SES to high	0.75	0.49	1.14
middle SES to high	1.34	0.90	2.00
visited SD/not visited (for those who did not visit GP)			
age ≥60			
low SES to high	1.01	0.75	1.34
middle SES to high	1.09	0.82	1.43
age<60			
low SES to high	0.44	0.35	0.55
middle SES to high	0.70	0.56	0.87

## 4. Discussion and Conclusion

The data from more than 28,000 respondents of the 2009 Israeli Health Survey was used to run log-linear models for categorical data analysis. These models described the main effects and interactions among characteristics of disadvantaged populations (SES, age, location and visits to GP and SD) regarding possible inequality in primary and secondary medical care utilization. The SES of respondents was determined based on their occupation, education level and by local socioeconomic indicators and housing conditions. For each respondent, medical care utilization was measured by a tally of visits to a GP (primary medical care, Model GP), to a SD (secondary medical care, Model SD) and visits to a SD conditioned on visits to a GP (primary and secondary medical care, Model SD/GP). The results enabled the following conclusions:

a) Elderly respondents displayed pro-poor utilization of primary medical care, with an odds ratio for this utilization more than twice as high, from low to high SES, in the periphery as opposed to the center;
b) Younger respondents displayed a lack of interaction between SES and primary medical care utilization;
c) Odds ratios for primary medical care utilization increased for elderly relative to younger respondents, and this increase was higher for respondents with low and middle SES than with high SES;
d) Elderly respondents displayed a lack of interaction between SES and secondary medical care utilization;
e) Younger respondents who did not visit a GP in the reported period (two weeks) showed pro-rich utilization of secondary medical care;
f) Among respondents who visited a GP in the reported period, the odds ratio for secondary medical care utilization, for elderly relative to younger respondents, increased as SES decreased.

Most of the results are in line with those of other studies of medical care utilization among disadvantaged populations in developed countries. Pro-poor inequality in visited GP and pro-rich inequality in visited SD has been reported in many studies of OECD countries that provide universal health care coverage (van Doorslaer et al., 2004; van Doorslaer et al., 2006; Bago d’Uva et al., 2009; Arendt, 2012).

In Norway, where universal health care coverage is provided, GP care is equally or pro-poor distributed, while specialist outpatient care is utilized more by higher SES populations (Hansen et al., 2012). Similar findings were reported for Ontario, Canada, where all necessary physician services are covered by the provincial government, without co-payments or deductibles (Glazier et al., 2009). In Japan, pro-rich inequality was observed among citizens from 20-64 years of age, but equal healthcare utilization was found among those 65 years and older (Watanabe & Hashimoto, 2012). For Japan, age was also found to be a factor that positively influenced utilization of preventive health care (Tsutsui et al., 2012). In Israel, it was found that “persons of low economic status were more likely than those of higher economic status to be high users of primary care services” and “were less likely to be high users of specialty care” (Shadmi et al., 2011).

In this report, the interactions of several disadvantage factors (age of 60+, location in the periphery, low SES) on the utilization of different types of medical care were estimated. Particularly, the odds ratios of visiting a SD for elderly people (comparing low to high SES and middle to high SES) were not consistent with their visits to a GP, as shown by comparing the first two rows in the first (Model GP) and second (Model SD) parts of Table 5. These groups of disadvantaged populations visit a SD with less frequency than what could be expected from their frequency of visits to a GP. However this might be explained by the chronic character of diseases typically found among elderly populations, which do not require frequent SD consultations due to a lack of new symptoms. On the other hand, the odds of younger respondents visiting a SD without visiting a GP were much lower for those with low and middle SES, as compared to respondents with high SES. That might be related to difficulty in accessing medical care during regular working hours. Therefore, the younger and working populations, especially ones with higher SES, might prefer to skip GP visits and make a direct appointment with a SD, even though some co-payments are deducted in certain cases. Using visits to a GP as an indicator of health status, it can be concluded that the status of elderly people with low SES, especially in the periphery, is lower than of those with high SES (Table 6, the first two rows).

The results of this article are in accord with predictions of the “health capital” investments model of Grossman (Grossman, 1972; 1999). Particularly, the first part of conclusion a) and conclusion c) of our study correspond to the predictions that, under reasonable assumptions on the cost and efficiency of health care investment, optimal health stock “falls with age,” while higher SES is “positively correlated with health,” reinforcing “the positive relationship between education and health.”

Visiting a SD without visiting a GP can result in an increased likelihood that the SD visit will be financed privately, and this in turn can demand decision making that often requires an advanced level of education. This can be explained within the framework of the previously mentioned model of health capital investments by Grossman.

The following policy implications can be drawn from this study:

a) Medical care policy can be differentiated for groups of disadvantaged populations by interactions of several important factors, which may include age, SES and geographical location.
b) In public clinics, availability of routine procedures (such as receiving prescriptions for chronic ailments, scheduling appointments for a SD or clarification of health care benefits) should be increased for elderly, low SES populations, not only through better access to phone and Internet services but also through wider access to nursing and junior medical staff. This will help to reduce overwork of GPs, particularly in peripheral regions.
c) More information should be provided to younger populations with low SES about preventive medicine and on the possible consequences of delayed visits to a SD, in order to help them to make better decisions on investing in their health from a young age. Having medical consultations available after regular working hours, making these appointments less challenging for younger and working populations, seems to be helpful.

The main limitations of the study were that data corresponding to health status, respondent income and origin of head of household were not able to be used, as the relevant questions were not included in the health survey questionnaire. In part, these issues were addressed by using visited/not-visited GP categories as a proxy for health status, and occupation and education level of heads of household as proxies for income and origin.

In addition to the results of this study, the research models used may be relevant for analysis of tertiary medical care as well. This could be the subject of a future study.
